# Development of an algorithm for automatic classification of right ventricle deformation patterns in arrhythmogenic right ventricular cardiomyopathy

**DOI:** 10.1111/echo.14671

**Published:** 2020-05-03

**Authors:** Marijn H. A. Groen, Laurens P. Bosman, Arco J. Teske, Thomas P. Mast, Karim Taha, Frebus J. Van Slochteren, Maarten J. Cramer, Pieter A. Doevendans, René van Es

**Affiliations:** ^1^ Division of Heart and Lungs Department of Cardiology University Medical Center Utrecht University of Utrecht Utrecht The Netherlands; ^2^ Netherlands Heart Institute Utrecht The Netherlands; ^3^ Department of Cardiology Catharina Hospital Eindhoven Eindhoven The Netherlands

**Keywords:** arrhythmogenic right ventricular cardiomyopathy, classification, computer algorithm, strain

## Abstract

**Background:**

Different disease stages of arrhythmogenic right ventricular cardiomyopathy (ARVC) can be identified by right ventricle (RV) longitudinal deformation (strain) patterns. This requires assessment of the onset of shortening, (systolic) peak strain, and postsystolic index, which is time‐consuming and prone to inter‐ and intra‐observer variability. The aim of this study was to design and validate an algorithm to automatically classify RV deformation patterns.

**Methods:**

We developed an algorithm based on specific local characteristics from the strain curves to detect the parameters required for classification. Determination of the onset of shortening by the algorithm was compared to manual determination by an experienced operator in a dataset containing 186 RV strain curves from 26 subjects carrying a pathogenic plakophilin‐2 (*PKP2*) mutation and 36 healthy subjects. Classification agreement between operator and algorithm was solely based on differences in onset shortening, as the remaining parameters required for classification of RV deformation patterns could be directly obtained from the strain curves.

**Results:**

The median difference between the onset of shortening determined by the experienced operator and by the automatic detector was 5.3 ms [inter‐quartile range (IQR) 2.7–8.6 ms]. 96% of the differences were within 1 time frame. Both methods correlated significantly with *ρ* = 0.97 (*P* < .001). For 26 *PKP2* mutation carriers, there was 100% agreement in classification between the algorithm and experienced operator.

**Conclusion:**

The determination of the onset of shortening by the experienced operator was comparable to the algorithm. Our computer algorithm seems a promising method for the automatic classification of RV deformation patterns. The algorithm is publicly available at the MathWorks File Exchange.

## 1 INTRODUCTION

Arrhythmogenic right ventricular cardiomyopathy (ARVC) is an inherited disorder characterized by progressive myocardial replacement by fibrofatty tissue; this predisposes patients to life‐threatening ventricular arrhythmias and dysfunction of the right ventricle (RV) predominantly.^1^, ^2^ The clinical diagnosis is based on the presence of ventricular arrhythmias, electrocardiographic and structural/functional abnormalities, combined with family history and the presence of ARVC‐associated mutations.^3^ These mutations are found in more than 60% of the ARVC patients, most frequently in the gene encoding the desmosomal protein plakophilin‐2 (*PKP2*).^4^ However, diagnosing ARVC is complex as these mutations are known to have incomplete penetrance and ARVC has a variable disease expression. Early manifestations of ARVC can be subtle and relatively asymptomatic, with sudden cardiac death (SCD) as first presentation.^5^ This emphasizes the importance of early diagnosis and detection of individuals at risk for life‐threatening arrhythmias.

**FIGURE 1:**
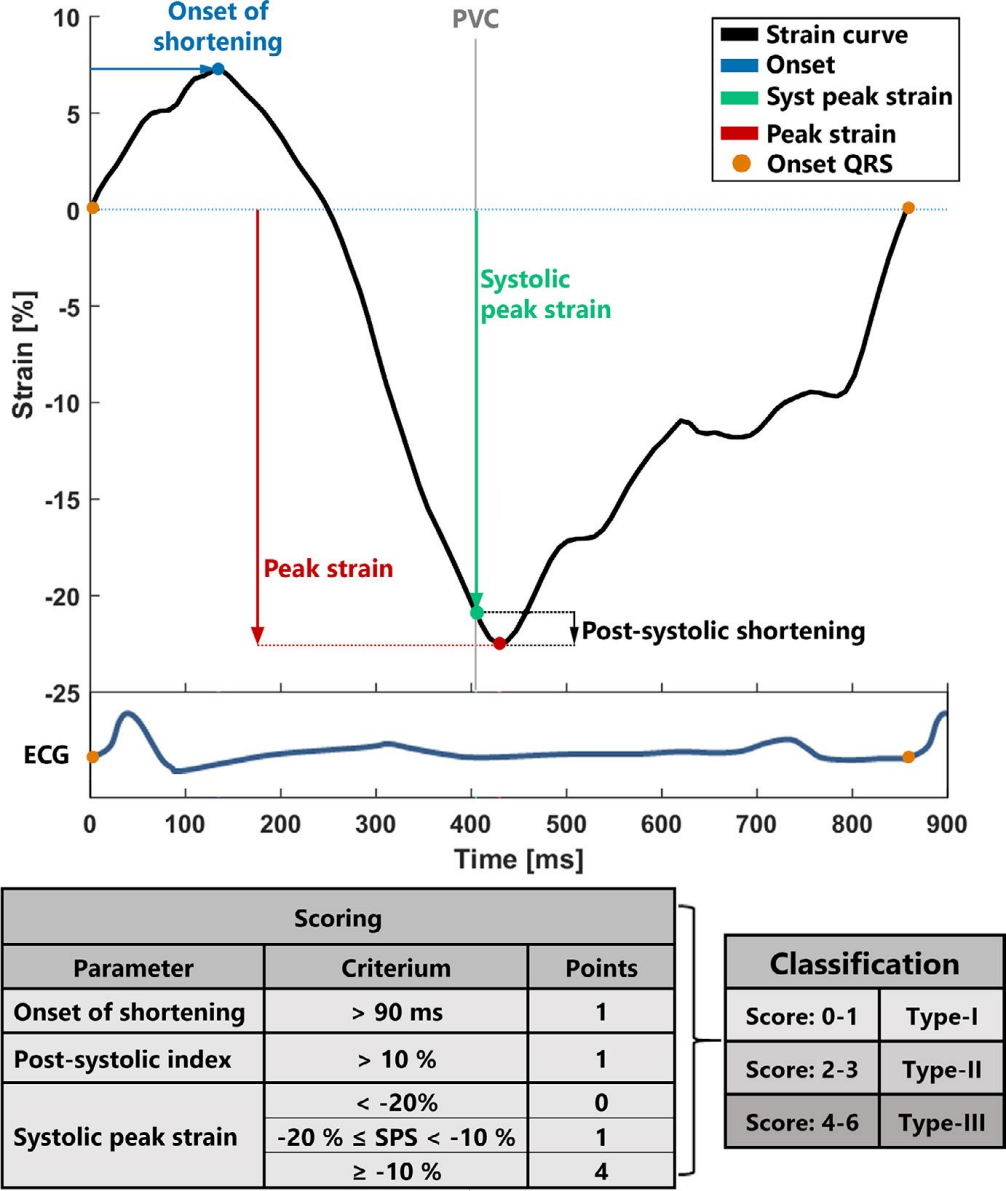
Example of a strain curve of the basal right ventricular segment, with the corresponding ECG. The onset of shortening (blue) = time between onset‐QRS (orange circle) and the onset of mechanical shortening. Systolic peak strain (green) is the maximal negative value between pulmonary valve opening and closure. Peak strain *(PS)* (red) is the maximal negative strain. Postsystolic shortening (black) is the peak strain minus the systolic peak strain and is used to calculate the postsystolic index, according to formula (Equation 1). In the lower table, the classification of the RV deformation pattern as defined by Mast et al^13^ is explained. Three parameters are used to score the deformation pattern: onset of shortening, postsystolic index, and systolic peak strain. Based on the scoring, the curves can be marked with the accompanying classification score. It is important to note that a systolic peak strain of ≥−10% directly results in a classification score of 4 points and thus type III classification. In comparison, type I classification corresponds to a normal deformation pattern, and type II shows a delayed timing of the onset of shortening, increased postsystolic index, and reduced systolic peak strain. ECG = electrocardiogram; PVC = pulmonary valve closure; SPS = systolic peak strain

In general, electrical abnormalities are considered to manifest before signs of structural disease, which has led to the recognition of different clinical stages of ARVC; (a) subclinical or concealed stage, when no electrocardiographic (ECG) or structural abnormalities (eg, regional contractile dysfunction and increased myocardial stiffness^6^) are present; (b) electrical, with solely ECG abnormalities; and (c) structural, with both ECG and structural abnormalities.^7^, ^8^ Interestingly, recent echocardiographic deformation (strain) imaging studies have demonstrated abnormal deformation patterns of the basal right ventricle (RV) myocardium in subclinical stages of ARVC, suggesting that sensitive assessment of mechanical function already shows structural dysfunction in earlier stages.^9^ This implies a potential role for RV strain analysis in detecting early disease and at‐risk individuals.^10^, ^11^, ^12^, ^13^ Consequently, deformation pattern analysis has recently been suggested by the European Association of Cardiovascular Imaging as part of the clinical evaluation for ARVC.^12^ Various abnormal strain parameters have been associated with ARVC pathology, including the delayed onset of mechanical shortening, (systolic) peak strain, and postsystolic shortening relative to the peak strain (postsystolic index; Figure 1). Combining these four parameters, Mast et al identified three types of deformation patterns related to the clinical ARVC stage (Figure 1).^13^, ^14^ Importantly, this deformation pattern classification method was shown to have added prognostic value for clinical disease progression during early stages of ARVC.^14^

Currently, the measurement of these parameters and classification of the strain pattern is an offline, manual process potentially prone to inter‐ and intra‐observer variability (or interpretation) which is directly related to experience level. The aim of this study was to eliminate these limitations, by designing an algorithm for fast, uniform, and automatic classification of RV deformation patterns and validating the performance.

## 2 METHODS

### 2.1 Population

Echocardiographic data were retrospectively obtained from 26 plakophilin‐2 (PKP2) mutation carries and 36 healthy control subjects that received echocardiography including deformation imaging in the University Medical Center Utrecht between 2006 and 2015. This study was approved by the local institutional ethics review board.

### 2.2 Imaging

Echocardiography was performed as previously described using a Vivid 7 or Vivid E9 ultrasound (US) machine (General Electric, Milwaukee, Wisconsin) using a broadband M3S transducer. The RV lateral free wall was visualized using the RV focused apical 4‐chamber view, after optimizing temporal resolution by reducing its sector width.^13^ Pulmonary valve opening and closure were determined using RV outflow tract spectral Doppler measurements during end‐expiration.

### 2.3 Image processing

Right ventricle (RV) strain curves were obtained using two‐dimensional strain imaging using standard B‐mode images (speckle‐tracking) as described before.^15^ With this technique the speckles as generated by the reflected US beam are followed frame by frame, resulting in a unique speckle pattern. Displacement of the speckle pattern represents the myocardial deformation. Images were analyzed using EchoPAC (version 12, GE Vingmed Ultrasound AS). Separate strain curves of the basal, mid‐ventricular, and apical segments of the RV free wall were obtained, composing a final dataset of 186 curves.^15^ The onset of the QRS complex was determined manually. Classification of the RV deformation patterns was performed manually using the strain curve of the RV basal lateral segment.^13^

### 2.4 Preprocessing

The saved strain curves were loaded into Matlab R2015a (MathWorks, Inc). All strain curves were interpolated to 1000 samples to create a uniform database with small sample intervals. That way, a precise comparison between the analysis of an experienced operator and the algorithm can be performed. The timing of the onset of the QRS complex was used as a reference.

### 2.5 Algorithm

There are four parameters required to calculate the classification; onset of shortening, peak strain, systolic peak strain, and the postsystolic index (Figure 1). These parameters were determined based on the following algorithm. First, strain curves without a peak systolic stain below −10% were scored with 4 points (Figure 1) and during classification of the basal lateral curves automatically marked as a type III ARVC class. These curves were excluded for analysis of the onset of shortening, as these are noninformative for validating the algorithm performance.

#### 2.5.1 Onset of shortening

**FIGURE 2:**
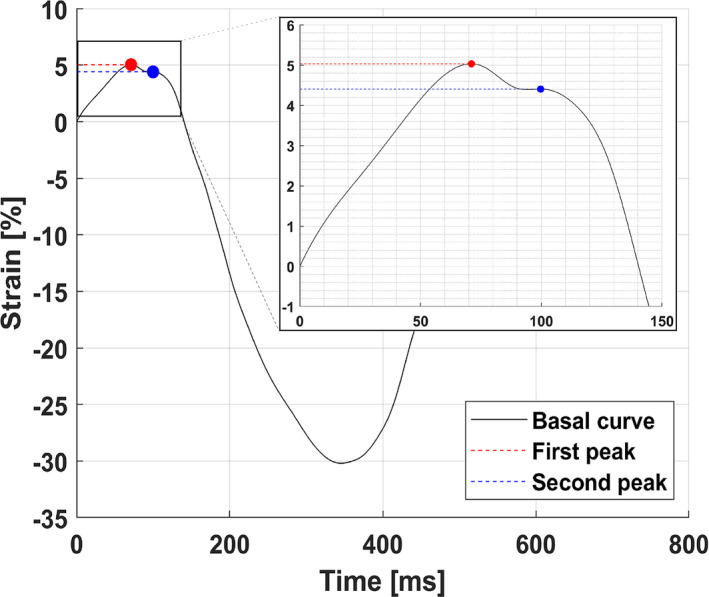
Example of a strain curve in which two peaks were detected in the first part of the curve. The dotted red line represents the first peak while the dotted blue line represents the second peak. The absolute difference between the two peaks is only 0.63%, and therefore, the onset of the mechanical shortening is set at the second peak

Onset of shortening is defined as the time between the onset of the QRS complex and the onset of the mechanical shortening measured from the strain curves (Figure 1). Assuming onset of the mechanical shortening always starts before pulmonary valve closure (PVC), only the segment of the strain curve between the onset of the QRS complex and PVC was analyzed. Onset of shortening was determined by comparison of the different peaks in the strain curve before PVC. A peak was defined as a local maximum, where the target data point is larger than both neighboring data points. If no peaks are detected before PVC, onset of shortening was assumed to be at the onset of the QRS complex. With one peak before PVC, the timing of this peak was stored as the onset of shortening. If two peaks are detected before PVC, the strain offset between these two peaks was compared (Figure 2). If the second peak was more than 1.5% absolute strain below the first peak, the timing of the first peak was used; otherwise, the timing of the second peak was used. This process was repeated for any subsequential peaks until one peak met the onset criteria.

#### 2.5.2 Peak strain

Peak strain is the maximal negative strain value and was determined using a MATLAB function which determines the minimal value of the curve.

#### 2.5.3 Systolic peak strain

The systolic peak strain was defined as the minimal strain value before or at the PVC, whichever was the lowest value. The systolic peak strain was determined using a MATLAB function which determines the minimal value of the curve between the start and the PVC.

#### 2.5.4 Postsystolic index (PSI)

The PSI was calculated using the previous determined peak strain and systolic peak strain using the following formula, which was implemented in the algorithm.

(1) $$\text{Post}\;\text{systolic index}\;=100∙\frac{\text{peak}\;\text{strain}\;-\;\text{systolic peak strain}}{\text{peak}\;\text{strain}}$$

If the peak strain is equal to the systolic peak strain, then PSI is set to zero.

After all parameters were calculated, all curves were scored and classified as type I, type II, or type III according to the criteria defined by Mast et al^13^ (Figure 1).

### 2.6 Validation

**FIGURE 3:**
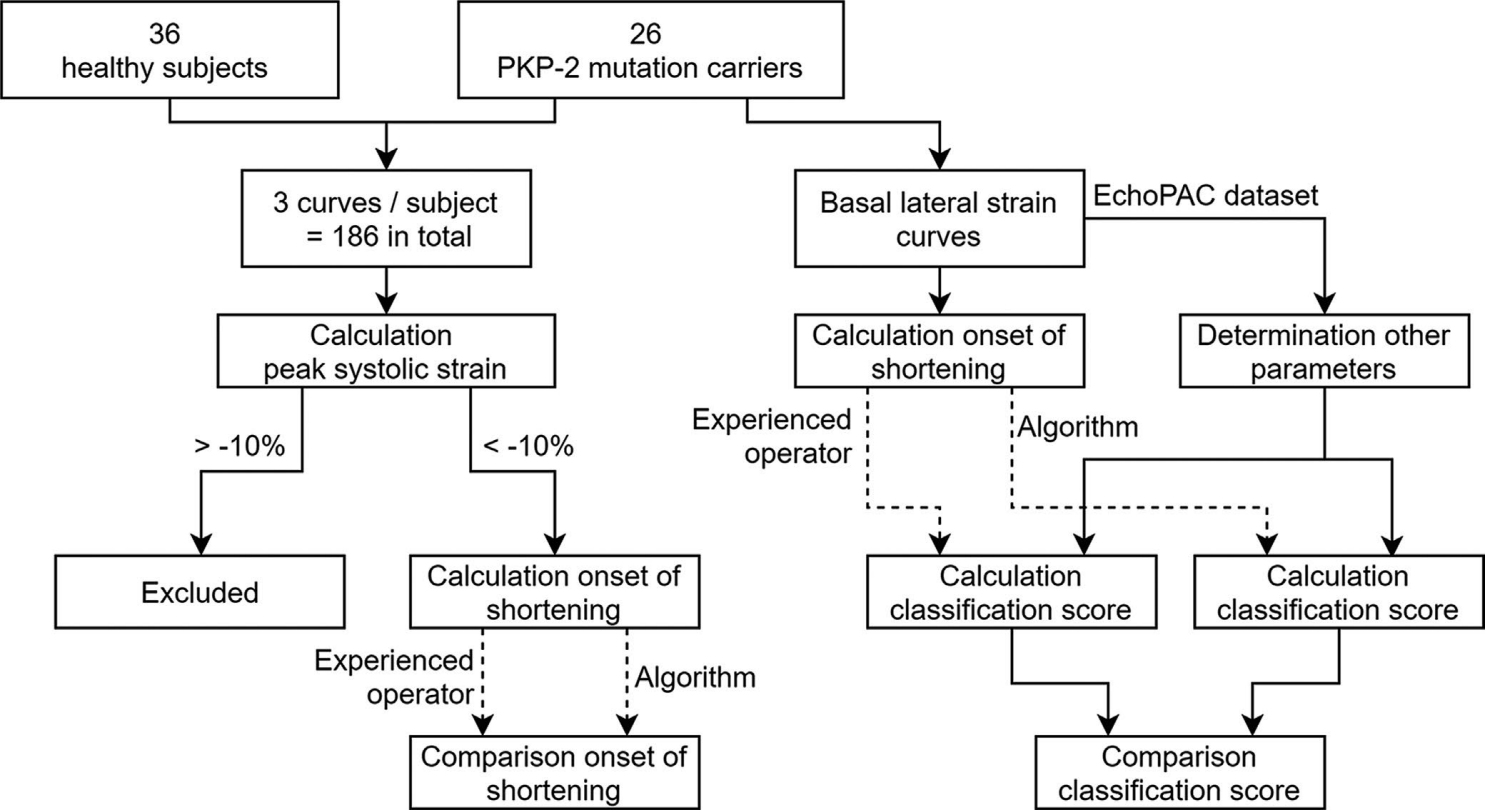
Flowchart of the calculation and validation method. For the comparison of the onset of shortening, both the three curves of the healthy subjects and the three curves of the *PKP2* mutation carriers were included. Curves with a peak systolic strain >−10% were excluded from the analysis. The basal lateral strain curves of *PKP2* mutation carriers were analyzed to compare the classification score between the algorithm and the experienced operator

The determination of the onset of shortening was validated using a specific validation procedure, as shown in the flowchart (Figure 3).

All 186 curves were evaluated by two experienced operators (AT and TM) independently, who manually determined the onset of shortening blinded from the algorithm outcome and the subject characteristics. The differences in absolute time were compared between the two operators, to give an example of inter‐operator differences. To validate the algorithm, both the differences in onset of shortening in absolute time and in timeframes (calculated by dividing the absolute difference in time by the sample time per curve) were compared between the first experienced operator (AT) and the algorithm.

Next, the basal lateral strain curves of the 26 *PKP2*‐mutation carriers were used to determine the classification type per subject and compare classification type between the algorithm and the first experienced operator (AT).

### 2.7 Statistical analysis

Values are presented as median and inter‐quartile range or mean ± SD as appropriate. Results are displayed using a Bland‐Altman graph. The onset of shortening as determined by algorithm is compared with the onset of shortening as determined by the first operator by calculating a (intra‐class) correlation coefficient. Groups were compared by an independent samples *t* test or Mann‐Whitney *U* test. Proportions were compared between groups using Fisher's exact test. *P*‐values of <.05 were considered significant.

## 3 RESULTS

### 3.1 Population

In this study, 36 healthy subjects and 26 subjects carrying the plakophilin‐2 (*PKP2)* mutation were included. Of the *PKP2* mutation carriers, 20 (77%) fulfilled definite ARVC diagnosis by the 2010 TFC. ARVC diagnosis was defined as fulfillment of ≥4 points by the revised 2010 Task Force Criteria.^3^ The baseline characteristics are provided in Table 1. *PKP2* carriers were older than control subjects (43.5 ± 16.1 years vs 36.0 ± 9.8, *P* = .028). *PKP2* carriers had significantly increased RV size and decreased LV/RV function by conventional echocardiographic measurements, compared to control subjects.

**TABLE 1:** Values are presented as mean ± SD or n (%)

	PKP2 (n = 26)	Controls (n = 36)	*P*‐value
Age (y)	43.5 ± 16.1	36.0 ± 9.8	.028
Male	13 (50)	17 (47)	.516
Probands	11 (42)	‐	‐
Anti‐arrhythmic medication	8 (31)	‐	‐
ICD	6 (23)	‐	‐
RV pacing	1 (4)	‐	‐
2010 Task Force Criteria			
Definite ARVC	20 (77)	‐	‐
Borderline ARVC	4 (15)	‐	‐
Possible ARVC	2 (8)	‐	‐
Echocardiography			
RV WMA	19 (73)	0 (0)	<.001
RVOT‐PLAX (mm)	34.0 ± 9.1	26.6 ± 4.7	<.001
RVOT‐PSAX (mm)	34.5 ± 9.1	29.1 ± 5.0	.005
RV FAC (%)	34.8 ± 10.5	44.4 ± 7.3	<.001
TAPSE (mm)	17.7 ± 4.3	24.1 ± 2.2	<.001
RV S’ velocity (cm/s)	9.8 ± 1.8	13.4 ± 2.6	<.001
LVEF (%)	56.8 ± 8.1	61.0 ± 5.3	.033

Note

*P*‐values of <.05 are considered significant.

ARVC = arrhythmogenic right ventricular cardiomyopathy; FAC = fractional area change; ICD = implantable cardioverter‐defibrillator; LVEF = left ventricular ejection fraction; PKP2 = plakophilin‐2; PLAX/PSAX = parasternal long‐/short‐axis view; RV = right ventricular; RVOT = right ventricular outflow tract; TAPSE = tricuspid annular plane systolic excursion; WMA = wall motion abnormality (akinesia, dyskinesia, or aneurysm).

### 3.2 Validation

For all 186 curves, the four parameters could successfully be determined by the algorithm within 0.4 seconds. Fourteen curves did not reach −10% peak strain and were therefore marked as type III and excluded from the validation of the onset of shortening.

### 3.3 Comparison onset of shortening

**FIGURE 4:**
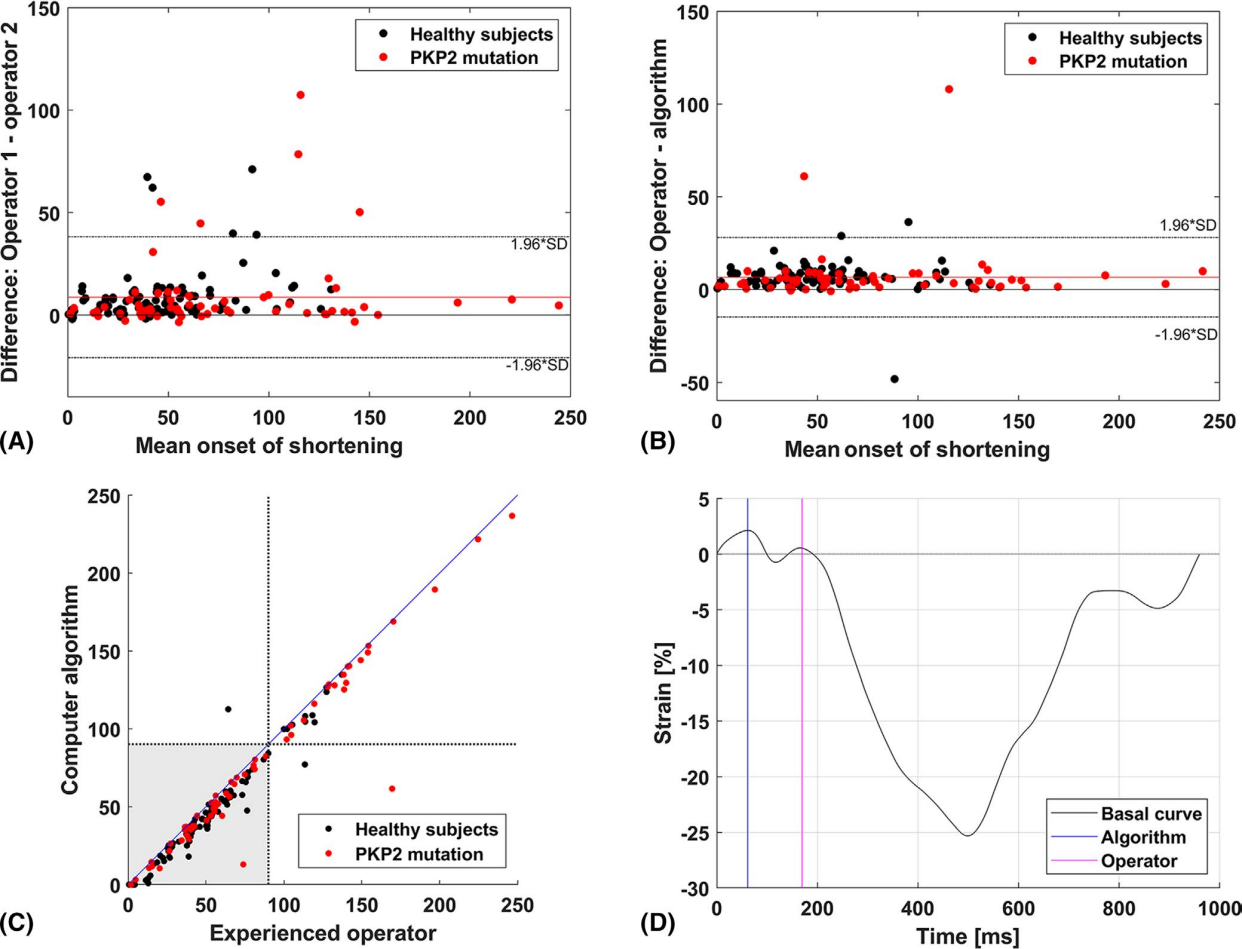
A, Bland‐Altman plot of the difference between the two experienced operators in onset of shortening. Black dots represent the healthy subjects while red dots represent the ARVC patients carrying a *PKP2* mutation. The red line represents the mean difference of 8.7 ± 15.0 ms B, Bland‐Altman plot of the difference between the first experienced operator and algorithm in onset of shortening. The red line represents the mean difference of 6.6 ± 10.9 ms The timing of the experienced operator is overall slightly later than the timing of the algorithm. C, Differences in onset of shortening between the algorithm and the first experienced operator. The two black dotted lines represent the threshold value for the classification as stated by Mast et al^13^ The lower‐left segment (gray) represents the curves with normal onset of shortening, while the upper‐right corner shows the delayed onset of shortening. Note that this difference does not distinguish between normal and abnormal strain curves, since onset of shortening is only one of the four parameters needed for classification. Both the upper‐left and the lower‐right corners represent the values where the algorithm and the experienced operator scored differently. The blue line represents the perfect linear relation (*x* = *y*) between the algorithm and the experienced operator. D, Example of the RV curve was the difference between the algorithm and the experienced operator showed the largest difference. In this case, two peaks were detected. The absolute difference in strain was 1.57%, and therefore, the first peak was marked as the onset of shortening by the algorithm

The median difference between the onset shortening determined by the two experienced operators was 4.3 ms (IQR 1.5–9.8 ms) (Figure 4A).

The algorithm was able to detect the onset of shortening directly in 148 curves (86.0%). In 24 curves (15 cases, 9 controls), a subsequent peak was selected by the automatic process previously described. The difference in onset of shortening time between the selected peak and the peak before that was −64 ± 31 ms For these cases, the median difference between the onset shortening determined by the algorithm and the first experienced operator was 3.5 ms (IQR 1.4–6.4 ms).

Overall, the median difference between the onset shortening determined by the algorithm and the first experienced operator was 5.3 ms (IQR 2.7–.6 ms) (Figure 4B). The correlation coefficient between the algorithm and the first experienced operator for the onset of shortening was *ρ* = 0.97 (*P* < .001), and the intra‐class correlation coefficient was 0.96 (0.94–0.97). Three curves (2%) were scored differently between the algorithm and the first experienced operator (Figure 4C, D). The mean framerate (which is depended on the settings during the US imaging) was 75 Hz, resulting in a duration of ~13 ms between two frames (time frame). 96% of the differences in all curves were within 1 time frame.

### 3.4 Comparison of the classification score

There were no differences in classification of the *PKP2* mutation carriers as determined by the algorithm and the experienced operator, based on differences in timing of the onset of shortening of the basal lateral curve.

## 4 DISCUSSION

In this study, an automatic algorithm was created for the analysis and classification of RV strain curves. The results suggest that the algorithm is capable of accurate detection of the four classification parameters and thereby capable of calculating the accompanying classification score. The results of this study show that our proposed automatic algorithm is feasible, quick, and applicable to nonexperts on RV deformation characteristics.

In this study, the operators have 13 and 3 years of experience in analyzing RV strain curves, respectively. The inter‐operator differences as shown in Figure 4A underscore the need for a more reliable method to determine the required strain parameters. As shown in Figure 4D, in some cases there was a large difference between the algorithm and the experienced operator due to the presence of multiple peaks in the first phase of the curve. In these cases the inter‐operator differences were equally large, because there is no strict consensus on which peak to use as the start of contraction. By using an algorithm, systematic analysis of the RV strain curves will result in uniform and reliable output.

A high (intra‐class) correlation was seen between the algorithm and the experienced operator for the onset of shortening. However, overall the onset of shortening as determined by the algorithm was slightly earlier than the operator, median of 5.3 ms (IQR 2.7–8.6 ms). An explanation for this difference might be that the operator visually picks the location on the curve at which the curve is clearly descending. The algorithm however is able to determine the ultimate first moment where the curve is descending and is therefore earlier than the operator. It is important to keep in mind that the minimum frame duration is 5 ms The median difference of 5.3 ms between the algorithm and the operator as found in this study is thus the same magnitude as the temporal resolution of the US machine.

The first step of the algorithm was to exclude all curves without strain below −10% and mark these curves as a type III ARVC class. In the scoring system described by Mast et al,^13^ the threshold for the systolic peak strain is −10%; every curve with a systolic peak strain ≥−10% was assigned 4 points. In the classification, 4‐6 points were classified as a type III and therefore it is valid to mark these curves as type III and not to score the onset and the amount of postsystolic shortening (which are both characteristically abnormal in the type III strain pattern).

Classification of the RV deformation patterns was based on the RV basal lateral segment, which is the subtricuspid region, since this region is typically first affected in ARVC.^10^, ^16^, ^17^ No differences were seen in classification type between the algorithm and the experienced operator. The assumption was made that peak strain, systolic peak strain, and the PSI did not differ between the algorithm and the experienced operator since the estimation of the peak strain, systolic peak strain, and the postsystolic index are straightforward and could be visually verified (eg, Figure 1).

### 4.1 Limitations

The classification by the algorithm was based on a single strain curve from the center of the basal lateral segment, while the classification method of Mast et al^13^ was based on the averaged parameters over the whole segment. It is unknown whether the classification will be different for these two methods. This should be studied during further research. Also, for the validation of the onset by the algorithm, all 3 segments (basal, mid‐ventricular, and apical) per patient were used instead of the basal segment only. Although we do not expect a significant interaction on the main results, these factors might have influenced the validation of the algorithm.

For the calculation of the inter‐operator difference, the analysis of two operators who work at the same clinical center was compared. The differences between operators of different centers might be even more significant, suggesting that the presented inter‐operator differences do not reflect real‐life values. However, this comparison falls out of the scope of our research question.

One patient had a DDD pacemaker and might have been paced in the RV during echocardiography. Although ventricular pacing may affect strain patterns, we do not expect that this affected the results of the algorithm.

### 4.2 Clinical relevance

Previous studies have shown that RV strain analysis is very useful for early detection and classification in ARVC*.* ^13^, ^14^ In those studies, the clinical relevance of the classification score as used in this algorithm was shown, as well as the prognostic value of RV strain analysis for disease progression in patients with ARVC. Our algorithm provides a simple and fast method to analyze the (RV) strain curves and to calculate the classification score. With the use of the algorithm, intra‐ and inter‐observer differences (based on variability of interpretation and experience level) will be abolished, paving the way for the clinical implementation of our 3‐step RV strain interpretation and classification even in nonacademic centers. The manual analysis and scoring of the strain curves are time‐consuming; using a computer algorithm will result in a relevant reduction of required time to perform the analysis. Both in a clinical setting as for research aims, the algorithm provides a method to compare data between different observers and different centers, thereby stimulating collaboration between different research groups.

The algorithm is vendor‐independent and is publicly available at the MathWorks File Exchange.^18^ Future steps to advance the clinical implementation would be to develop an easy‐to‐access online tool. Moreover, to stimulate clinical use of (RV) strain analysis, this type of algorithms should be implemented in the postprocessing software of the different vendors.

In this study, we focused on the classification of deformation patterns of the RV in ARVC patients. However, this method is not limited to solely RV strain in this specific patient population, but might be useful for several other myocardial diseases which have a distinct fibrosis pattern,^19^ left bundle branch block, and, for example, ischemic heart diseases. Future research could use our algorithm for the analysis of deformation patterns in these other diseases.

## 5 CONCLUSION

In this study, an automatic algorithm was developed and verified to automatically analyze RV deformation patterns. Using this algorithm, intra‐ and inter‐observer differences are prevented, resulting in fast and uniform analysis of strain curves. Specialistic laboratories are working on RV strain analysis in ARVC for more than ten years; this algorithm might stimulate the implementation of their methods into the real world.

## CONFLICTS OF INTEREST

None.
